# Impacts of an Oral Appliance on Snoring in Adults with Varying Degrees of Snoring Severity: A Preliminary Study

**DOI:** 10.3390/medicina61050893

**Published:** 2025-05-14

**Authors:** Yu-Hsiang Cheng, Jui-Kun Chiang, Yen-Chang Lin, Hsueh-Hsin Kao, Yee-Hsin Kao

**Affiliations:** 1 Department of Finance, Shih Hsin University, No. 1, Ln. 17, Sec. 1, Muzha Road, Wenshan Dist., Taipei City 116, Taiwan; yuhsiang@mail.shu.edu.tw; 2 Department of Family Medicine, Dalin Tzu Chi Hospital, Buddhist Tzu Chi Medical Foundation, No. 2, Minsheng Road, Dalin, Chiayi 622, Taiwan; roma@tzuchi.com.tw; 3 Nature Dental Clinic, Puli Township, Nantou 540, Taiwan; drlin@alliswell.tw; 4 Department of Radiation Oncology, Taichung Veterans General Hospital, Taichung 407, Taiwan; 5 Department of Family Medicine, Tainan Municipal Hospital (Managed by Show Chwan Medical Care Corporation), 670 Chung-Te Road, Tainan 701, Taiwan

**Keywords:** oral appliance, snoring, obstructive sleep apnea (OSA), sleep

## Abstract

*Background and Objectives*: Oral appliances (OAs) are commonly used to manage sleep-disordered breathing conditions, including primary snoring, and offer an alternative treatment for individuals with obstructive sleep apnea (OSA) who cannot tolerate continuous positive airway pressure (CPAP) therapy. Our study analyzed the possible factors associated with higher snoring rates compared with those associated with lower snoring rates. *Materials and Methods*: A customized dental brace with a tongue compressor was the essential part of the Lin OA (LOA). The compressor is available in various lengths, ranging from 0.5 to 3.0 cm across different versions. The participants wore the LOA throughout the night while sleeping. Their snoring rates were recorded using the SnoreClock app on their cell phones. *Results*: The analysis included 36 participants, comprising 30 males and 6 females. The participants had a mean age of 44.91 ± 9.96 years, a mean BMI of 26.18 ± 3.50 kg/m^2^, and an average recording duration of 398.27 ± 77.56 min per session. In total, 4052 sleep recordings were analyzed. The number of files for females was less than that for males (563 vs. 3489). In this study, individuals belonging to the highest one-third based on the baseline snoring rate (H group) experienced a significant reduction in snoring, approximately 84.8%, when using the LOA-3 cm device equipped with a 3 cm tongue compressor. The individuals who belonged to the lower two-thirds based on the baseline snoring rate were classified as the L group. Among male participants, snoring decreased by approximately 66.4%. Similarly, among female participants in the L group, snoring decreased by approximately 69.3% when using the LOA-3 cm. *Conclusions*: In this study, we observed a significant reduction in snoring for the two groups of participants wearing the LOA-3 cm, with the rate decrements ranging from 66.4% to 84.8%. This reduction was more pronounced in the H group than in the male participants in the L group. Further studies are needed to explore the reasons for these findings.

## 1. Introduction

Snoring, characterized by snorting or grunting sounds during sleep, results from the vibration of soft tissues in the upper airway [1]. While it can be a nuisance for bed partners, snoring is often associated with obstructive sleep apnea syndrome (OSAS) and various chronic diseases [2]. The prevalence of snoring in females is lower than that in males in Taiwan (42.5% vs. 60.8%) [3], Japan (20% vs. 43%) [4], the U.S. (25% vs. 42%) [5], Turkey (8.9% vs. 29.5%) [6], and Korea (8.4% vs. 15.6%) [7]. One reason for the varying prevalence of snoring may be that people of Chinese descent tend to have narrower cranial bases and flatter midface structures than people of other ethnic groups [8]. A previous meta-analysis reported a significantly higher prevalence of snoring in men, with a combined odds ratio of 1.89 compared to women [9]. Additionally, variations in snoring prevalence may be influenced by the age of the population and characteristics such as BMI, smoking, and neck circumference [10].

Occasional snoring is almost universal. However, there is evidence suggesting that snoring itself may be harmful. Snoring has been recognized as a key risk factor for hypertension, diabetes, and cardiovascular disease (see [11,12,13,14,15]): the louder the snoring, the higher the risk of developing hypertension [16]. A previous study reported that the intensity of snoring rises as obstructive sleep apnea (OSA) becomes more severe [17]. Another previous study reported that snorers have twice the risk of carotid stenosis compared with non-snorers [18]. It is believed that the activation of the sympathetic nervous system plays a role in the elevated cardiovascular risk associated with OSA [19]. Previous studies have also reported that snoring can elevate sympathetic activity, as evidenced by changes in breathing frequency, inspiratory and expiratory times, and tidal volume in response to snoring or simulated snoring through high-frequency oscillations [20,21,22]. These findings suggest that snoring affects the autonomic nervous system, increasing sympathetic nervous system activity. Building on previous findings, detecting snoring and providing appropriate interventions could improve the health of affected patients.

Sleeping position adjustments are recommended for most snorers before interventions. Advanced treatments include the use of mandibular advancement devices, surgical options, and continuous positive airway pressure ventilators [23]. Consequently, oral appliances (OAs) are nowadays recommended for individuals with primary snoring [24]. Traditional OAs enhance upper airway function by positioning the mandible anteriorly. This mechanism intends to prevent airway collapse during sleep [25]. The traditional appliances previously mentioned have various names, including mandibular advancement devices (MADs), mandibular repositioning appliances (MRAs), and mandibular advancement splints (MASs) [26]. Customized OAs are created using physical impressions of a patient’s unique oral structures [27]. Studies show that customized, titratable OAs can lower scores on the visual analog scale, the apnea–hypopnea index (AHI), and the Epworth sleepiness scale. Additionally, they can decrease arousal and oxygen desaturation while increasing oxygen saturation to a greater extent than non-customized, titratable OAs [24,28].

**Figure 1:**
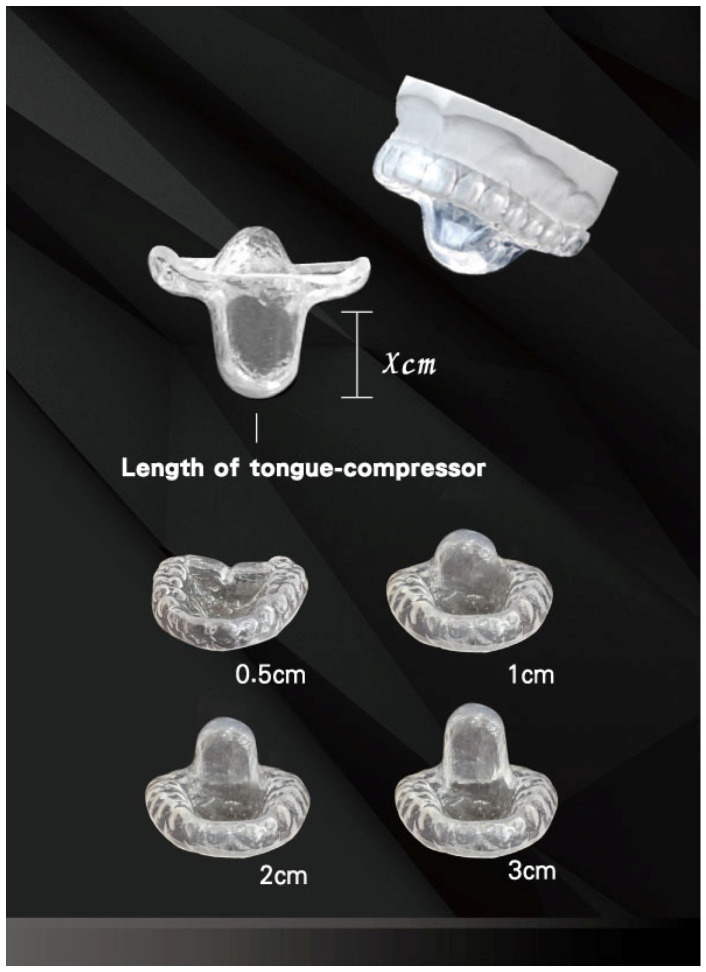
The various tongue compressor lengths of the LOA.

Oral appliances have been shown to decrease both the occurrence and severity of snoring and to enhance patients’ sleep quality and quality of life [24]. This study evaluates the effect of a tongue-compressing oral appliance in snorers, a device type with limited models currently available in clinical practice. The Lin Oral Appliance (LOA) is a customized device designed to compress the tongue by different lengths across its versions (Figure 1). Patients might visit the dentist for reasons beyond snoring, such as temporomandibular (TM) joint discomfort, acid reflux, asthma, myofascial pain, insomnia, anxiety, or other related concerns; therefore, we designed this study to evaluate the effectiveness of the LOA in patients with varying degrees of snoring severity. The SnoreClock app (iOS version 1.8.0 and Android version 3.8.0.) recorded snoring sounds to determine snoring rates.

## 2. Materials and Methods

### 2.1. Study Design

We conducted an observational study to evaluate the effect of a novel oral appliance, the Lin OA, on snoring rates in adult patients. The study protocol was reviewed and approved by the Research Ethics Committee of the Buddhist Dalin Tzu Chi General Hospital in Chiayi, Taiwan (Nos. B10901020 and B10703013).

### 2.2. Study Population

In this study, we expand on our previous research by incorporating new cases and employing alternative analytical methods [29]. A total of forty-one patients experiencing snoring issues were recruited from a dental clinic in Taiwan between 1 October 2018 and 31 October 2021.

### 2.3. Interventions

We provided each patient with an LOA (patent number I602555 [Taiwan], ZL 2013 1 0753192.9 [China]) [30]. The LOA consisted of one customized dental brace and a fixed tongue compressor, with the length of the compressor varying based on the version (0.5, 1, 2, or 3 cm) but with a standard thickness of 0.5 cm. The LOA was developed in 2012 by Dr. Yen-Chang Lin. As a fully customized device, it is crafted using dental technology to match an individual’s unique dental structure. The material used is a common dental material, soft ethylene vinyl acetate, a non-toxic and environmentally friendly compound made from the copolymerization of ethylene and vinyl acetate. The LOA is worn on the upper jaw and extends downward to form a structure that applies pressure to the tongue. The operating principles of the LOA include the following: First, by utilizing different lengths of the tongue compressor, the LOA helps maintain a patent upper airway and expands the oro-laryngopharyngeal space when the wearer is sleeping in the supine position. Second, the support provided by the tongue compressor increases the surface tension of the oropharyngeal structures, thereby reducing tissue vibration and the incidence of snoring. Third, the tongue compressor aids in supporting the harmonized fascial system, which interpenetrates and surrounds muscles, organs, and other structures, creating an integrated environment that promotes the coordinated function of the whole-body system [31].

The patients voluntarily participated and signed informed consent forms prior to their enrollment in this study. Demographic details, such as gender, age, body mass index, and medical history, were gathered. Participants were also given clear instructions on the correct use of the LOA. The educational program informed participants that, based on their adaptation to the device, they could switch from an LOA with a shorter length to one with a longer length. Furthermore, the potential benefits of various versions in reducing snoring were discussed. On-site demonstrations were provided to ensure all patients understood how to use the LOA before participating.

We used the SnoreClock smartphone app to record the snoring rates of patients at home due to its accuracy, accessibility, and user-friendliness [32]. The participants downloaded the SnoreClock app onto their smartphones by themselves. The SnoreClock software (iOS version 1.8.0 and Android version 3.8.0.) was manufactured by Ralph-Rüdiger Schiffhauer. Patients were instructed to keep their smartphones within 30 cm of the head of their bed but were not given specific restrictions regarding phone locations; for example, patients could put the devices on a bedside table or on the bed. After completing the required recordings during their full night’s sleep each night, the patients gave their smartphones to our researchers for subsequent analysis. All research procedures were conducted following the approved study protocol.

### 2.4. Outcome Measures

The objective of this study was to assess the effectiveness of the LOA in reducing snoring rates among adult patients with varying levels of snoring severity.

### 2.5. Statistical Analysis

R (version 4.4.2), a free software product supported by the “R Foundation for Statistical Computing” located in Vienna, Austria, was used to perform statistical analyses. The frequencies of categorical variables were computed to summarize the data. The means and standard deviations of continuous variables (mean ± standard deviation) are also provided. To test the difference between the means of a continuous variable for the two groups, we used Student’s *t*-test. A two-sample proportion test was applied to test differences in proportions between the two groups. In addition, we performed a *t*-test to analyze the significance of each variable in the regression model. The statistical significance level was set at 0.05.

To explore the impact of the LOA version and other characteristics on snoring rates, this study considered two types of linear models: a pooled regression model and a random effect model. Let Y be the snoring rate and $X_{1},\cdots ,X_{p}$ denote the lengths of the LOA and other potential characteristics; a linear model can then be expressed as

$$Y\approx f\left(X_{1},\cdots ,X_{p}\right),$$

where f is a linear function. The snoring rate ranges from 0 to 1; however, the predicted snoring rate values obtained using the above model are not guaranteed to range from 0 to 1, which is undesirable. A common strategy for solving this problem is to transform Y so that the transformed variable has no range constraint. Here, we consider the logit transform, which gives the following model:

$$log\left(Y/(1-Y)\right)\approx g\left(X_{1},\cdots ,X_{p}\right),$$

where g is a linear function. After estimating the coefficients in *g*, we can obtain the estimate of g, denoted by $g^{*}$. Therefore, the predicted value of Y, denoted by $Y^{*}$, can be expressed as follows:

$$Y^{*}=\frac{exp\;(g^{*}(X_{1},\cdots ,X_{p}))}{1+exp\;(g^{*}(X_{1},\cdots ,X_{p}))}$$

## 3. Results

**Figure 2:**
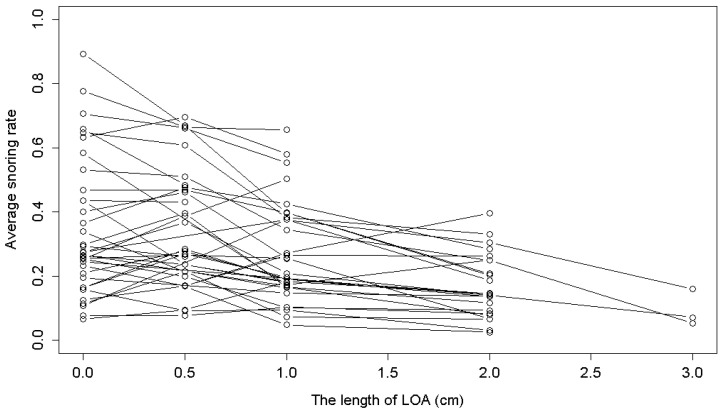
Mean snoring rates of enrolled participants based on different lengths of the LOA.

The original dataset included observations of snoring rates, LOA versions used, and other measurements for the 41 participants. After excluding 1 participant who used the same version of the LOA throughout the observation period and 4 participants who lacked baseline snoring rates (snoring rates when not using the LOA), 36 participants (30 men and 6 women) were enrolled in this study and contributed a total of 4052 recordings. The snoring rate was defined as the proportion of snoring time out of the total sleep time, as outlined in [33]. Figure 2 shows the average snoring rates for participants using different versions of the Lin Oral Appliance. From the figure, we can observe that the snoring rates of some participants appear to be lower when using a longer LOA; however, for other participants, the snoring rates show a trend of first increasing and then decreasing as the LOA length increases. Indeed, we observed that for participants with moderate-to-low baseline snoring rates, the snoring rates were not always reduced when the participants used longer LOAs. Based on these observations, it was reasonable to first cluster the participants into different groups based on their baseline snoring rates before further analyzing the impact of LOA usage on snoring rates.

The participants were divided into H and L groups according to their baseline snoring rates (the snoring rates of each participant were retrieved while not using the LOA). Participants with baseline snoring rates greater than the 66.7th percentile of the baseline snoring rates were placed in the H group, while the rest of the participants (with baseline snoring rates less than or equal to the 66.7th percentile of the baseline snoring rates) were placed in the L group. The sizes of the two groups were quite different: the H group consisted of 12 participants with 1201 records, and the L group consisted of 24 participants with 2851 records, as shown in Table 1. Table 1 also shows a significant difference in recording time between the two groups of participants: the mean recording time for the L group was approximately 10 min longer than that of the H group. In contrast, the differences in BMI (body mass index) and age between the two groups were not significant.

**Figure 3:**
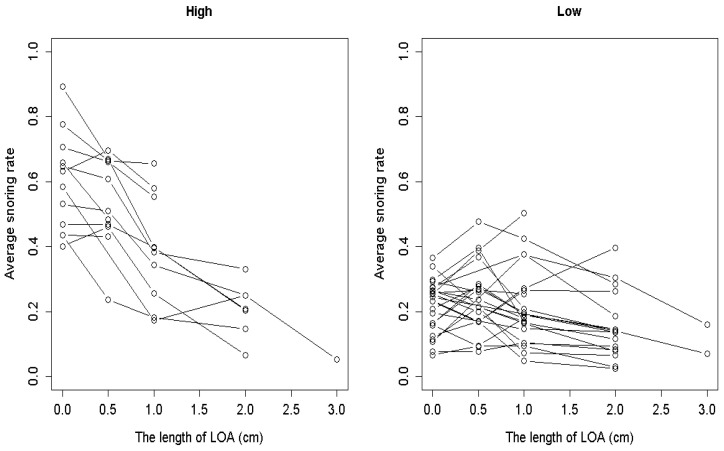
Mean snoring rates of participants in the H and L groups based on different versions of the Lin Oral Appliance.

Figure 3 shows the average snoring rates for the H and L groups for the different versions of the LOA, and the corresponding summary statistics are provided in Table 1. The *p*-values in Table 1 indicate a significant difference in snoring rates between the two groups of participants for different LOA versions (LOA-0, LOA-0.5, LOA-1, LOA-2). Here, LOA-0 denotes the cases without the LOA.

To show the effects of using LOAs with different lengths on snoring rates and the differences in snoring rates between the two groups of participants, we present in Table 2 the changes in average snoring rates when the participants used the next version of the LOA. The number in the top row in each cell represents the average change in snoring rate when switching from a shorter to a longer LOA version. Most of the changes are negative and significantly less than zero (see *p*-value ^b^). The *p*-values for the bottom row are not available due to insufficient observations for those cases. We found that the snoring rate generally decreased as the LOA length increased, except for the change from LOA-0 to LOA-0.5 in the L group. For the L group’s version 0 to version 0.5 case, the average change was 2.16%, which is not significantly different from zero (see *p*-value ^c^). We also compared the average changes between the H group and the L group for each version switch, and the difference was significant for version 0 to 0.5 and version 0.5 to 1 (see *p*-value ^a^). In summary, for the H group, there was a significant decrease in snoring rates for the 0 to 0.5, 0.5 to 1, and 1 to 2 version switches, while for the L group, there was a significant decrease in snoring rates only for the 0.5 to 1 and 1 to 2 version switches. Note that for the L group, even though the average change in snoring rates was positive for the 0 to 0.5 version switch, the increase was not significant. Additionally, the improvements in snoring rates in the H group were greater than those in the L group for the 0 to 0.5 and 0.5 to 1 version switches.

**Figure 4:**
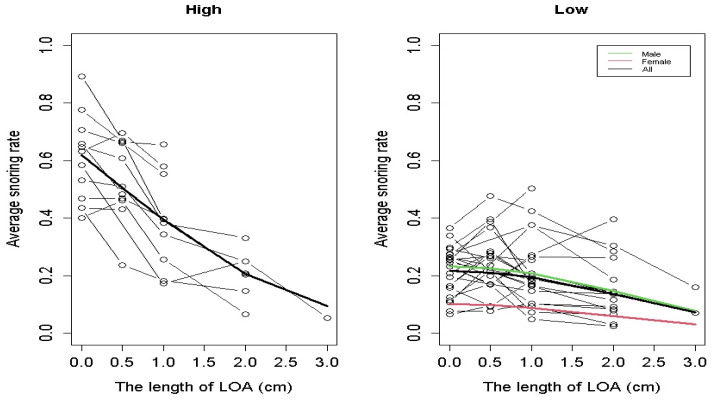
Estimated trends in the average snoring rates for the H and L groups. In the right panel, the red and green curves indicate the estimated trends for females and males, respectively, based on Model 1. The black curve illustrates the estimated trend based on Model 2, which did not contain gender as a factor.

In this study, we applied pooling and random effect regression models to both the L and H groups. Several potential explanatory variables were considered, including LOA length, LOA length squared, BMI, age, number of LOA usage days, and gender. We used a forward procedure for variable selection to determine the final model; the variables included in the final model are shown in the upper half of Table 3. Table 3 presents the coefficient estimates for the pooled regression model and the random effect model. We used the Lagrange Multiplier test to determine which model (pooled model or random effect model) was more suitable for modeling the data in the H group and the L group, and the resulting *p*-values indicate that the random effect model was better for both groups. The estimated trends in the average snoring rate for the H and L groups are shown in Figure 4, and the predicted snoring rate values for different LOA lengths based on the random effect regression model are presented in Table 4. The results in the upper half of Table 3 suggest that in the H group, only LOA length significantly affected the snoring rate, with its influence trend depicted by the thick black curve on the left side of Figure 4. In contrast, for the L group (see Model 1), both LOA length squared and gender were found to be significant predictors of the snoring rate. These effects are illustrated by the thick green curve (for males) and the red curve (for females) on the right side of Figure 4. Due to the relatively small number of female participants in the L group, we removed the gender variable from the random effect regression model and the pooled regression model while keeping the LOA length squared variable in the models; the results of coefficient estimates are presented in the lower half of Table 3 (Model 2). The impact of LOA length squared on the snoring rate is shown by the thick black curve on the right side of Figure 4. This study proposes two models for the L group, based on two considerations: the univariate regression results identifying gender as a significant factor (see Appendix A) and the relatively small number of female participants.

Figure 4 illustrates the estimated trends in average snoring rates for the H group and the L group, while Table 4 presents the corresponding predicted snoring rates (%) for different LOA lengths in both groups based on a random effect model. For the H group, there is an obvious decreasing pattern in the average snoring rate as the LOA length increases, and the reductions in snoring rates as the LOA length increases by one centimeter are about the same for different LOA versions, as shown in Table 4 and the left panel in Figure 4. For the L group, participants were further divided into male and female subgroups, referred to as L group-male and L group-female, respectively, as gender was a contributing factor in the random effects model; however, gender was not a significant factor in the H group. Table 4 presents the predicted snoring rates for the two subgroups, and the right panel of Figure 4 shows two curves of estimated trends in the average snoring rates for males and females in the L group. The right panel of Figure 4 shows that the snoring rate curve for males is higher than that for females at each LOA length. For both curves, there is a decreasing trend when the LOA length exceeds 1 cm, and the decreasing rates of the snoring rate curve for the male subgroup are slightly greater than those for the female subgroup. Moreover, the performance of Model 2 for the L group is highly similar to that for the male subgroup in the L group, as shown in both Figure 4 and Table 4.

In summary, for participants in the H group, the predicted snoring rate decreased from 61.9% when not wearing the LOA to 50.6%, 39.3%, 20.6%, and 9.4% when wearing LOA-0.5 cm, LOA-1 cm, LOA-2 cm, and LOA-3 cm, respectively. The corresponding approximate improvement rates are 18.3%, 36.5%, 66.7%, and 84.8% [(61.9 − 9.4)/61.9], respectively. For participants in the L group, the predicted snoring rate decreased from 23.2% to 22.5%, 20.7%, 14.7%, and 7.8% for males and from 10.1% to 9.8%, 8.9%, 6.0%, and 3.1% for females, respectively. The corresponding approximate improvement rates are 3.0%, 10.8%, 36.6%, and 66.4% [(23.2 − 7.8)/23.2 = 15.4/23.2] for males and 3.0%, 11.9%, 40.6%, and 69.3% [(10.1 − 3.1)/10.1 = 7/10.1] for females, respectively. This study found that participants may have stopped using the longer versions of the LOA after using LOA-2 cm, as significant improvements in snoring rates were observed.

## 4. Discussion

Participants in the highest one-third with regard to snoring rates (H group) experienced an 84.8% reduction in snoring with the LOA-3 cm. Among male participants in the lower two-thirds (L group), snoring decreased by 66.4%, while female participants in the L group saw a 69.3% reduction in snoring rates. Overall, all participants experienced a significant reduction in snoring, with a decrease of at least 66.4% when using the LOA-3 cm. This study recommends using the LOA to improve snoring, suggesting that participants gradually progress to the LOA-3 cm version for optimal results. It may take approximately 3 to 6 months to transition to the LOA-3 cm version and achieve the desired outcomes. However, the exact timeframe was not systematically documented, which is a limitation of this study.

We also observed a trend of decreasing snoring rates as the length of the oral appliance increased in both the H and L groups. However, there are noticeable differences in how the two groups respond to changes in snoring rates. Participants with a high baseline snoring rate typically experience an immediate reduction in snoring upon starting to use the oral appliance. For participants with a low baseline snoring rate, using an LOA (0.5 cm version) may initially show an insignificant increasing trend, followed by a significant reduction in the snoring rate. This phenomenon could be attributed to the fact that this subgroup of patients may not primarily use the LOA to address snoring issues but rather for other conditions, such as insomnia, TM joint discomfort, acid reflux, myofascial pain, anxiety, allergies (particularly those affecting the respiratory system or skin), and other related factors.

Additionally, for participants with low baseline snoring rates, snoring rates are influenced by gender, whereas no such gender effect is observed for participants with high baseline snoring rates. The model established based on these subgroup distinctions helps explain why some participants experienced no improvement in snoring rates when starting with a 0.5 cm appliance. Studies have identified male gender, older age, and higher BMI as common risk factors for snoring [34,35]. In this study, the severity of snoring was found to be higher in females than in males. However, the sample variance for the average snoring rates for females was 0.05009, which was nearly twice the sample variance of the average snoring rates for males. This large variation may stem from only six female participants being included.

As mentioned in [29], tongue compression by the LOA may effectively keep the upper airway open, increase oropharyngeal volume during supine sleep, and reduce oropharyngeal vibration. Therefore, the LOA is equipped with some mechanisms aimed at reducing snoring.

Additionally, this study used the SnoreClock smartphone application to measure participants’ snoring rates, allowing for the accurate collection of continuous data on participants’ snoring rates as they slept at home [32]. To compare the effectiveness of the LOA and traditional appliances, future research should be conducted under the same environmental and measurement conditions. Furthermore, this study found that participants with different snoring rates exhibited varying changes in their snoring rates when using the LOA. Whether such a phenomenon still occurs when participants use traditional oral appliances is also an interesting issue that remains to be investigated.

This study did not examine whether participants had obstructive sleep apnea (OSA). Although oral appliances are commonly used for patients with primary snoring, they are also increasingly used to treat OSA [24]. Some studies have confirmed that using oral appliances (OAs) to treat OSA patients can reduce snoring rates [27,36,37]. However, the measurement of snoring rates in these studies was often based on the patient’s subjective assessment, such as the Basic Nordic Sleep Questionnaire [38]. Additionally, past studies did not account for the differences in snoring rate changes due to the use of OAs by different groups of participants. Therefore, in future research, we suggest investigating the effect of the LOA and OAs on reducing the snoring rate in OSA patients in a controlled environment and taking accurate measurements. Whether response patterns vary across different subject groups should also be investigated.

In a previous study, SnoreClock was identified as a highly accurate app for detecting snoring [32]. Additionally, a prior meta-regression analysis reported a strong correlation between snoring severity and the apnea–hypopnea index (AHI) in studies utilizing polysomnography (PSG) [33]. However, the snoring rate derived from SnoreClock is not regarded as the gold standard for assessing the AHI, which is established using PSG. In this study, participants visited the dentist due to varying degrees of snoring. However, polysomnography (PSG) was not performed prior to using the LOA, which represents a limitation of this study.

Although this study provides information regarding the effectiveness of the LOA in reducing snoring rates in different groups, it shares the same limitations as [29]. First, sleep quality indices such as the oxygen desaturation index and apnea–hypopnea index were not included in this study. These may be important factors associated with snoring rates. Additionally, the number of participants was limited, particularly regarding females. This could have been because the data were collected over several months of sleep recordings, and fewer female snorers in Taiwan were willing to participate in the treatment. Measures should be implemented to address this problem in future research.

## 5. Conclusions

In this study, participants in the highest one-third with regard to baseline snoring rates experienced an 84.8% reduction in snoring when using the LOA-3 cm version compared with not using an LOA. Among male participants with moderate-to-low baseline snoring rates, snoring decreased by 66.4%, while female participants in the same group experienced a 69.3% reduction. This study confirms that the LOA can help alleviate snoring and that participants can achieve optimal results by continuing its use up to the 3 cm LOA version. However, fewer female snorers were willing to participate in this treatment. Measures should be taken in future research to address this issue. This study also suggests that, for participants with moderate-to-low baseline snoring rates, using LOA versions with lengths of 1 cm or above is expected to help reduce snoring, even if using shorter versions does not. Further studies using larger datasets are needed to better understand the underlying causes of these findings.

## Figures and Tables

**Table 1:** Demographics and characteristics of the enrolled participants.

Variables	All	H Group	L Group	*p*-Value
Participants	36	12	24	
Number of records	4052	1201	2851	
Female/male	6/30	4/8	2/22	0.155
Age, years	44.91 ± 9.96	42.83 ± 10.96	46.15 ± 9.38	0.393
BMI, kg/m^2^	26.18 ± 3.50	26.01 ± 3.38	26.27 ± 3.65	0.859
Recording time, min	398.27 ± 77.56	391.77 ± 68.96	401.01 ± 80.77	<0.001
Snoring rate, % [n *]				
LOA-0	29.48 ± 22.43 [532]	56.53 ± 15.71 [144]	19.44 ± 15.02 [388]	<0.001
LOA-0.5 cm	31.76 ± 21.41 [430]	54.97 ± 18.37 [117]	23.08 ± 15.07 [313]	<0.001
LOA-1 cm	25.87 ± 18.07 [1227]	33.21 ± 18.67 [574]	19.41 ± 14.79 [653]	<0.001
LOA-2 cm	15.22 ± 12.73 [1499]	21.75 ± 13.20 [356]	13.19 ± 11.88 [1143]	<0.001
LOA-3 cm	9.03 ± 8.82 [364]	5.38 ± 5.92 [10]	9.13 ± 8.87 [354]	0.080

* n: number of snoring records; H group: participants whose baseline snoring rates exceed the 66.7th percentile; L group: all other participants.

**Table 2:** Average changes in snoring rates (%) when switching LOA versions.

Version (cm)	All	H Group	L Group	*p*-Value ^a^
0 to 0.5	−0.83 (0.314 ^b^)	−6.27 (0.032 ^b^)	2.16 (0.235 ^c^)	0.0274
0.5 to 1	−7.20 (<0.001 ^b^)	−13.62 (<0.001 ^b^)	−4.15 (0.036 ^b^)	0.0182
1 to 2	−6.06 (<0.001 ^b^)	−9.69 (0.024 ^b^)	−4.47 (0.010 ^b^)	0.2520
2 to 3	−13.70	−19.65	−10.72	-

*p*-value ^a^: the difference is significant for version 0 to 0.5 and for version 0.5 to 1 switches; *p*-value ^b^: most of the changes are negative and significantly less than zero; *p*-value ^c^: for the L group’s version 0 to 0.5 case, the average change is 2.16%, which is not significantly different from zero; H group: participants whose baseline snoring rates exceed the 66.7th percentile; L group: all other participants.

**Table 3:** The coefficient estimates for the pooled regression and random effect regression models.

Group	Model	LOA Length	Squared LOA Length	Male vs. Female	Intercept	*p*-Value
H group						<0.001
	Pooled	−1.009 (<0.001)			0.536 (<0.001)	
	Random effect	−0.916 (<0.001)			0.483 (0.006)	
L group (Model 1)						<0.001
	Pooled		−0.129 (<0.001)	0.983 (<0.001)	−2.202 (<0.001)	
	Random effect		−0.141 (<0.001)	0.986 (0.004)	−2.186 (<0.001)	
L group (Model 2)						<0.001
	Pooled		−0.128 (0.002)	-	−1.312 (<0.001)	
	Random effect		−0.142 (<0.001)	-	−1.282 (<0.001)	

H group: participants whose baseline snoring rates exceed the 66.7th percentile; L group: all other participants.

**Table 4:** Predicted snoring rate values (%) for different LOA lengths and group conditions based on the random effect regression model.

	LOA-0	LOA-0.5	LOA-1	LOA-2	LOA-3
H group	61.9	50.6	39.3	20.6	9.4
L group (model 2)	21.7	21.1	19.4	13.6	7.2
L group (model 1, male)	23.2	22.5	20.7	14.7	7.8
L group (model 1, female)	10.1	9.8	8.9	6.0	3.1

H group: participants whose baseline snoring rates exceed the 66.7th percentile; L group: all other participants.

## Data Availability

The data generated during and/or analyzed in this study are not publicly available but can be obtained by request from the corresponding author, Y.-H.K.

## Appendix A

**Table A1:** Univariate regression analyses for squared LOA length and gender in the L group.

L Group	Model	Intercept	Coefficient
Squared LOA length	Pooled	−1.312 (<0.001)	−0.128 (0.002)
	Random effect	−1.282 (<0.001)	−0.142 (<0.001)
Male	Pooled	−2.371 (<0.001)	0.981 (<0.001)
	Random effect	−1.364 (<0.001)	−0.003 (0.007)
